# Effect of preoperative vitamin D deficiency on functional outcomes after high tibial osteotomy: a retrospective case control study

**DOI:** 10.1186/s12891-020-03295-1

**Published:** 2020-04-17

**Authors:** Wonchul Choi, Jae Hwa Kim, Seong-Eun Byun, Han-Seung Ryu, David Rojas

**Affiliations:** 1 Department of Orthopaedic Surgery, CHA Bundang Medical Center, CHA University, 59, Yatap-ro, Bundang-gu, Seongnam-si, Gyeonggi-do Republic of Korea; 2 Department of Orthopaedic Surgery, Denver Health Medical Center, Denver, CO USA

**Keywords:** High tibial osteotomy, Medial open wedge high tibial osteotomy, Osteoarthritis, Vitamin D, 25-hydroxy vitamin D, Functional outcome

## Abstract

**Background:**

This study aimed to evaluate the effects of vitamin D deficiency on the functional outcomes following a high tibial osteotomy (HTO).

**Methods:**

Clinical data of 209 patients (327 knees) who underwent HTO done by a single surgeon were retrospectively registered for the study. Ninety-four patients (94 knees) who underwent HTO were analyzed. Patients were assigned into two groups according to their preoperative serum vitamin D levels (D- Deficiency and S- Sufficient); < 20 ng/ml (group D, *N* = 48) and ≥ 20 ng/ml (group S, *N* = 46) respectively. A subjective form of International Knee Documentation Committee (IKDC) score, mechanical alignment, and cartilage status before and 1 year following HTO were studied between groups.

**Results:**

The mean postoperative IKDC score was significantly higher in group S (*p* = 0.012). Moreover, the difference of IKDC score between pre- and post- HTO was also significantly higher in group S (*p* = 0.006). Preoperative vitamin D level and IKDC score did not show a significant correlation. Serum vitamin D level was found to be moderately correlated to postoperative IKDC score (*r* = 0.342 and, *p* = 0.001). A moderately positive correlation between serum vitamin D level and improvement of IKDC score following osteotomy was appreciated (*r* = 0.381 and, *p* < 0.001).

**Conclusion:**

Patients with vitamin D deficiency had less satisfactory functional outcomes within 1 year from HTO surgery. Our results suggest that preoperative serum vitamin D level is one of the factors affecting the outcome after HTO. We recommended analyzing serum vitamin D levels as part of the routine workup in patients undergoing HTO.

## Background

Vitamin D is an essential element that helps regulate and maintain bone homeostasis, and is therefore associated in various musculoskeletal disorders. The role of vitamin D in the progression of osteoarthritis (OA) has been recognized through an upregulation of vitamin D receptor in degenerated cartilage [1, 2]. High prevalence of vitamin D deficiency in patients with knee OA and it’s progression has also been described [3, 4]. Moreover, the impact of vitamin D deficiency on functional outcomes following arthroplasty in OA patients is also reported [5, 6].

Medial open wedge high tibial osteotomy (HTO) has been performed as the treatment option for relatively young and active patients with an isolated medial compartment OA with varus deformity [7, 8]. It has been suggested that vitamin D deficiency has a negative effect on outcomes following total knee arthroplasty (TKA) [6, 9], while the effects of vitamin D levels on functional outcomes in patients undergoing HTO has not been reported.

The main objective of this study was to evaluate the effect of vitamin D deficiency on the functional outcome of HTO by comparing (1) postoperative the subjective form of International Knee Documentation Committee (IKDC) score and (2) the changes in the the subjective form of IKDC score before and after HTO between patients with and without vitamin D deficiency.

We hypothesized that patients with vitamin D deficiency would show worse functional outcomes compared to those without vitamin D deficiency following HTO.

## Methods

### Study population and grouping

Following institutional review board approval, clinical data of 209 patients (327 knees) who underwent HTO done by a single surgeon (senior author) were retrospectively reviewed. To minimize bias, only the right side of patients who underwent HTO bilaterally was included in the analysis. Therefore, included for analysis were 94 patients who underwent medial open wedge HTO for primary medial compartment OA, and those with complete preoperative vitamin D level reports. All patients included for the analysis were followed for a minimum of 1 year from surgery and underwent a second look arthroscopic examination. Patients were allocated into two groups; group D (vitamin D-deficient) and group S (vitamin D-sufficient); according to the preoperative serum 25-hydroxyvitamin D level. Serum vitamin D level of 20 ng/ml was selected as a cut-off value according to the guideline [10, 11].

### Surgical technique and rehabilitation

All surgical procedures were carried out by a single surgeon. The patient was placed in the supine position with spinal or epidural anesthesia and a thigh tourniquet was inflated during the surgery. Knee arthroscopy is performed first to inspect and repair any concomitant menisci and/or ligamentous injuries inside the knee if necessary. Biplanar medial open wedge HTO is performed under fluoroscopic control according to the method that previously reported. Osteotomy is performed using osteotomes and a calibrated distractor used to open the osteotomy site to achieve the target mFTA; 3° valgus of mFTA; as preoperatively planned. Final Fixation of osteotomy is achieved using an anatomical locking plate (OhtoFix; Ohtomedical Co. Ltd., Goyang, Korea). After plate fixation, 5 cc of β-TCP (EXCELOS inject; CG Bio, Seongnam, Korea) (Ca_3_(PO_4_)_2_) is then injected into the osteotomy gap.

Patients were encouraged to start passive range of knee motion and active quadriceps strengthening exercises the day after surgery with a hinged knee brace protection. Partial weight bearing with crutches and brace were maintained for 4 weeks, followed by full weight bearing as tolerated.

HTO was staged with a minimum interval of 6 months in patients undergoing bilateral uni-compartmental OA to facilitate rehabilitation.

### Radiologic and clinical parameters

Differences in limb alignment and angle correction were compared. Radiographic parameters were measured with the picture archiving and communication system (PACS) using the standing hip-knee-ankle radiographs with the patella facing forward taken preoperatively and at 1-year-follow up. Differences in demographic factors (age, sex, and body mass index, etc.) were also compared between groups to find any potential confounders. Functional outcome assessment was performed utilizing the subjective form of IKDC score preoperatively and 1 year after HTO surgery. The degree of cartilage degeneration was also evaluated during initial and during a second-look arthroscopic examination. The status of cartilage was classified from grade 0 to grade 4 with reference to Outerbridge classification [12].

### Measurement of serum vitamin D, calcium, and albumin levels

Blood sample was collected in the morning after an overnight fast within 2 weeks before surgery, as a part of the preoperative work up at our institution. The serum concentration of vitamin D was measured by chemiluminescent immunoassay using ADVIA Centaur® Vitamin D Total assay (Siemens Healthcare Diagnostics Inc., Tarrytown, NY, USA). Serum calcium levels were measured through a Roche calcium gen.2 manual - absorbance assay (Roche Diagnostics System, Switzerland) using a COBAS c 702 module with CAPSO 557 mmol/L, NM-BAPTA 2 mmol/L (pH 10), EDTA 7.5 mmol/L (pH 7.3). Serum albumin levels were measured through Roche ALB2 manual - colorimetric assay (Roche Diagnostics System, Switzerland), using a COBAS c 702 module with citrate buffer 95 nmol/L (pH 4.1) and bromcresol green 0.66 mmol/L.

### Statistical analyses

All continuous variables were tested for normality using the Shapiro-Wilk test. Continuous variables are presented as means with standard deviations and ranges. A t-test was used for comparing continuous variables showing normality such as serum calcium, albumin, and postoperative IKDC scores between vitamin D-deficient and vitamin D-sufficient groups. Continuous variables without a normal distribution were analysed using the Mann-Whitney test between groups. Chi-square test was applied to compare discrete variables, including sex ratio and Outerbridge classification between groups. Spearman correlation analysis was performed to evaluate the association between serum vitamin D levels and postoperative IKDC scores, and the change of IKDC scores after HTO, since these variables did not show normality. *P* value < 0.05 indicates statistical significance. SPSS version 24.0 for Windows (IBM, NY) was used for all statistical analyses, except for our power analysis which was calculated using G*Power version 3.1.9.

A sample size was not previously determined due to the retrospective nature of the study which may limit the power analysis. However, a post hoc power analysis showed that this study had an 80.3% power to detect a significant difference between groups (Group-D versus Group-S), assuming an alpha error level of 5% and change of IKDC scores after surgery as the primary dependent variable.

## Results

Forty-eight knees (51.1%) were assigned to group D, while 46 knees (48.9%) were assigned to group S. The average serum vitamin D level was 14.1 ± 3.9 ng/mL (group D) and 28.1 ± 7.1 ng/mL (group S), respectively (*P* < 0.001). Besides the serum vitamin D serum level difference between groups, there was no statistical differences in serum calcium and albumin levels between groups (Table 1).

**Table 1:** Preoperative laboratory test of patients underwent high tibial osteotomy included in the current study

Variables	Total (*n* = 94)	Vitamin D-deficient group (*n* = 48)	Vitamin D-sufficient group (*n* = 46)	*P*-value
Serum 25 (OH) D (ng/mL)	21.0 ± 9.0	14.1 ± 3.9	28.1 ± 7.1	< 0.001
Serum calcium (mg/dL)	9.2 ± 0.7	9.2 ± 0.4	9.2 ± 0.9	n.s.
Serum albumin (g/dL)	4.5 ± 0.5	4.5 ± 0.3	4.6 ± 0.7	n.s.

*n.s.* nonsignificant

*P value< 0.05: considered significant. The values presented as the mean and the standard deviation

Mean age was similar between groups (58.0 years in group D and 59.7 years in group S). Other preoperative characteristics in each group did not show significant differences (Table 2).

**Table 2:** Demographics and preoperative characteristics of patients underwent high tibial osteotomy included in the current study

Variables	Total (*n* = 94)	Vitamin D-deficient group (*n* = 48)	Vitamin D-sufficient group (*n* = 46)	*p* value
Mean age, year	58.8 ± 8.1	58.0 ± 8.1	59.7 ± 8.0	n.s.
Sex, n (M/F)	33/61	21/27	12/34	n.s.
BMI, kg/m^2^	26.4 ± 3.7	26.4 ± 4.1	26.5 ± 3.4	n.s.
Preoperative mechanical alignment (°, varus)	5.9 ± 2.9	5.5 ± 2.9	6.3 ± 2.9	n.s.
Preoperative IKDC score	31.8 ± 14.0	31.8 ± 14.2	31.9 ± 13.9	n.s.
Outerbridge classification (no.)				
1	2	1	1	n.s.
2	18	8	10	
3	25	11	14	
4	49	28	21	

*n.s.* nonsignificant

*P value< 0.05: considered significant. The values presented as the mean and the standard deviation

The mean value of postoperative mechanical alignment was valgus 3.3° in group D and 2.7° in group S, without a statistical difference. Angle correction between groups did not show any significant difference as well.

Preoperative IKDC scores were similar between groups; however, postoperative IKDC scores were significantly higher in group S (45.8 vs. 53.3, *P* = 0.012), and the difference between IKDC scores before and after HTO was also significantly higher in group S (14.0 vs. 21.4, *P* = 0.006) (Tables 2 and 3).

**Table 3:** Comparison of postoperative outcomes between patients with and without vitamin D insufficiency

Variables	Total (*n* = 94)	Vitamin D-deficient group (*n* = 48)	Vitamin D-sufficient group (*n* = 46)	*P*-value
Postoperative IKDC score	49.4 ± 13.6	45.8 ± 13.8	53.3 ± 13.1	0.012
Change of IKDC score after operation	17.6 ± 13.2	14.0 ± 12.4	21.4 ± 13.0	0.006
Postoperative mechanical alignment (°, valgus)	3.0 ± 2.7	3.3 ± 2.5	2.7 ± 3.0	n.s.
Correction of angle (°)	8.9 ± 3.0	8.8 ± 3.3	9.0 ± 2.8	n.s.
Outerbridge classification (no.)				
1	9	4	5	n.s.
2	22	9	13	
3	31	19	12	
4	32	16	16	

*n.s.* nonsignificant

*P value< 0.05: considered significant. The values presented as the mean and the standard deviation

**Fig. 1:**
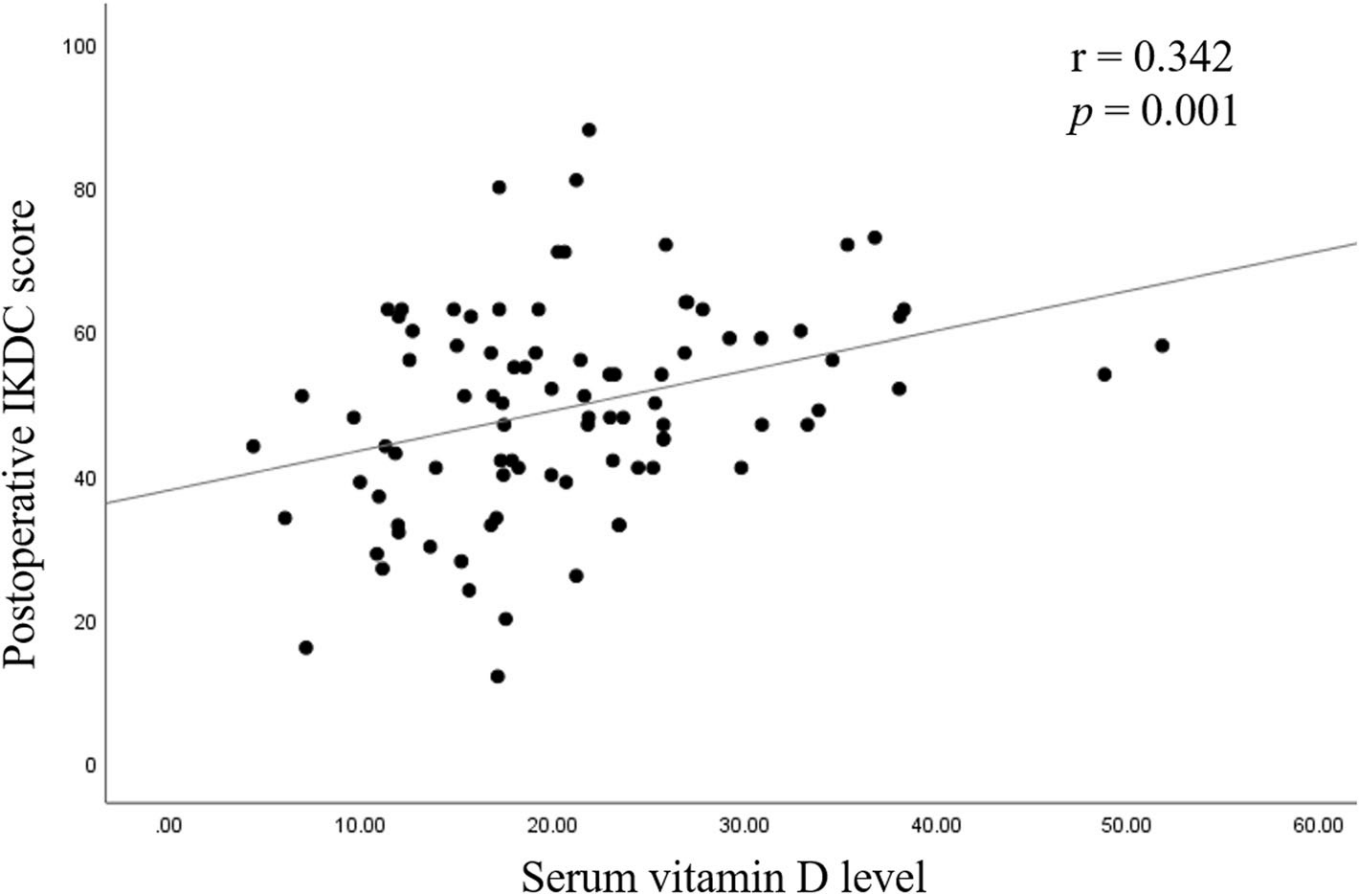
A scatter plot illustrating the relationship between serum vitamin D level and postoperative International Knee Documentation Committee (IKDC) score. A correlation analysis showed a moderate positive coefficient of 0.342

**Fig. 2:**
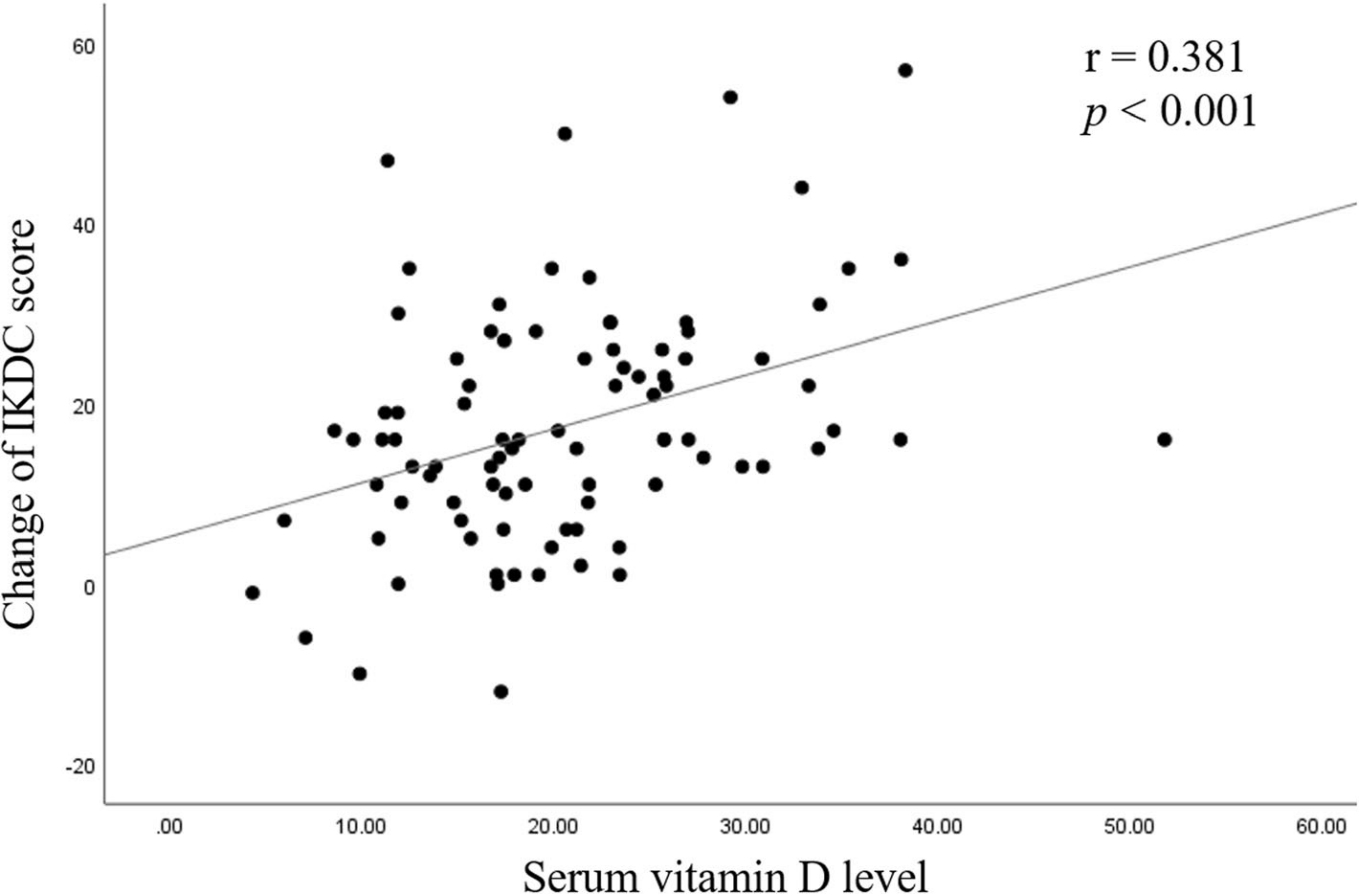
A scatter plot illustrating the correlation between serum vitamin D and change of International Knee Documentation Committee (IKDC) score after high tibial osteotomy. A correlation analysis showed a moderate positive coefficient of 0.381

Serum vitamin D level and preoperative IKDC scores did not show a significant correlation. Serum vitamin D level was found to be weakly correlated to postoperative IKDC scores (*r* = 0.342 and, *P* = 0.001; Fig. 1). A weakly positive correlation was found between serum vitamin D level and improvement of IKDC scores after HTO surgery (*r* = 0.381 and, *P* < 0.001; Fig. 2).

## Discussion

The main finding of the current study shows that patients with vitamin D deficiency had less satisfactory postoperative functional outcomes at 1 year following HTO compared to patients without vitamin D deficiency.

Recent studies demonstrate that vitamin D deficiency is a risk factor for less satisfactory functional outcomes following hip and knee arthroplasty procedures [5, 6]. Shin et al. reported that functional outcomes including Knee Society Score, alternative step test, and the six-meter walk test 3 month after TKA was significantly worse in patents with vitamin D deficiency than in those without vitamin D deficiency [6]. Nawabi et al. also reported significantly lower Harris Hip Score (HHS) in patients with vitamin D deficiency, and found a correlation between serum vitamin D levels and HHS scores preoperative and postoperative [5].

Skeletal muscle pathology caused by vitamin D deficiency is a suggested cause of worse functional outcomes after arthroplasty in vitamin D deficient patients. Vitamin D directly affects development and regeneration of skeletal muscle through vitamin D receptor (VDR) in skeletal muscle [13, 14]. In addition to muscle atrophy and impaired muscle regeneration, altered intramuscular calcium transport caused by vitamin D deficiency is also presumed to affect muscle biology and function [15]. Vitamin D regulates calcium transport by gene expression and non-transcriptional pathways inducing the activation of several transmembrane signals via VDR [16, 17]. Decreased grip strength and reduced calcium in mitochondria and sarcoplasmic reticulum has been demonstrated in vitamin D deficient animals [18, 19]. Physical performance, which is closely related with muscle function, has also been associated with vitamin D levels in humans [18, 20–22].

In patients undergoing HTO, cartilage, as well as muscle status can affect overall functional outcomes, since HTO preserves articular cartilage. Vitamin D deficiency accelerates degradation of the articular cartilage by inducing transforming growth factor- β, matrix metalloproteinases (MMP) -9 and MMP-13 [2, 23]. The association between vitamin D deficiency and decreased cartilage thickness has also been described [24]. While, protective effects of vitamin D supplementation on knee OA has been suggested [23]. Therefore, functional outcomes of patients undergoing HTO can be closely related with vitamin D deficiency. As expected, our results demonstrated that functional outcomes in patients undergoing HTO can be associated with serum vitamin D levels.

In our study, preoperative IKDC scores and cartilage status did not show a significant difference between groups (vitamin D; deficient and sufficient). In regard to preoperative IKDC scores and vitamin D levels, no correlation was found. Our results differ in the association between vitamin D and OA, as previously documented [24–26]. Indications of HTO could be a possible cause of this results. Since HTO is performed in relatively young and active patients with mild OA, patients with HTO tend to have similar preoperative functional and cartilaginous status regardless of their serum vitamin D level.

In the current study, about 51% of patients who underwent HTO had vitamin D deficiency. This high incidence of vitamin D deficiency in orthopaedic patients has also been noted in other studies looking at vitamin D levels, and using a similar a cut-off value of 20 ng/ml or less [27, 28].

The current study has limitations of note. First, the analysis was performed in a retrospective manner; therefore, selection bias could have affected our results. And, for the same reason, informed consent could not be obtained. However, all parameters including serum vitamin D levels were included through a routine protocol, and the data was collected prospectively, which minimizes retrospective analysis bias. Second, the research subject is not a representative cohort of population in a specific region, which can cause a bias. However, the bias could be minimized due to the small size and ethnically identical population of the country. Third, only subjective form of IKDC score was available to assess functional outcome, which can also bring bias to the current study. The subjective form of IKDC has been reported to be a useful tool for evaluating functional status objectively, including sports activity as well as symptoms in multiple knee joint pathologies [29, 30]. Therefore, in the authors’ opinion, the subjective form of IKDC score was sufficient for comparing functional outcome. Fourth, only preoperative level of vitamin D was assessed in the current study. Therefore, a possible change in vitamin D levels after HTO and the effect in outcomes could not be assessed. Lastly, markers of bone metabolism including serum PTH was not analyzed. However, since the purpose of the current study is to evaluate the effect of serum vitamin D level on postoperative outcome after HTO, in authors’ opinion, the impact of this limitation in not significant. And no difference in serum calcium and albumin level between groups may represents similar calcium homeostasis in both groups.

## Conclusion

Patients with lower preoperative serum vitamin D level showed less satisfactory functional outcomes at 1 year following HTO surgery. Our result suggests that preoperative serum vitamin D level is one of the factors affecting functional outcomes after HTO. Special attention to Vitamin D levels must be taken during perioperative work-up in patients undergoing HTO surgery.

## Data Availability

The datasets used and/or analysed during the current study are available from the corresponding author on reasonable request.
